# Strong-ties and weak-ties rationalities: toward a mental model of the consequences of kinship intensity

**DOI:** 10.3389/fpsyg.2024.1476018

**Published:** 2024-11-13

**Authors:** Kuang-Hui Yeh, Jane Terpstra Tong, Rachel Sing-Kiat Ting, Michael Harris Bond, Meetu Khosla, Virendra Pratap Yadav, Shashwat Shukla, Charles Liu, Louise Sundararajan

**Affiliations:** ^1^Academia Sinica, Taipei, Taiwan; ^2^School of Business, Monash University Malaysia, Subang Jaya, Malaysia; ^3^Department of Psychology, Jeffrey Cheah School of Medicine and Health Sciences, Monash University Malaysia, Subang Jaya, Malaysia; ^4^Department of Management and Marketing, Faculty of Business, Hong Kong Polytechnic University, Kowloon, Hong Kong SAR, China; ^5^Department of Psychology, Daulat Ram College, University of Delhi, New Delhi, India; ^6^Department of Applied Psychology, Shyama Prasad Mukherji College for Women, University of Delhi, New Delhi, India; ^7^Department of Management, University of Allahabad, Prayagraj, India; ^8^School of Psychology, Counseling, and Family Therapy, Wheaton College, Wheaton, IL, United States; ^9^Independent Researcher, Rochester, NY, United States

**Keywords:** kinship intensity, strong ties and weak ties, rationality, ecological niche, values and beliefs, habitus

## Abstract

There is growing evidence of the connection between variations in kinship intensity and cross-cultural differences in psychological traits. Contributing to this literature on kinship intensity, we put forward a mental model to explain the enduring connection between ancestral niche and psychological traits. Our model posits that two primary orientations or dispositions—strong-ties and weak-ties rationalities—have co-evolved with our ancestral niches to perpetuate—by internalizing and reproducing—the social structure (such as preferences for certain attitudes, values, and beliefs) of the ancestral niche. The findings from 1,291 participants across four societies—China, India, Taiwan, and the United States—support our hypothesis that strong-ties (weak-ties) rationalities, when activated, will endorse strong-tie (weak-ties) values and beliefs. This proposed model contributes to the toolbox of cultural and cross-cultural psychology in a twofold sense: First, in addition to the index of kinship intensity, it offers a measure of kin-based rationality as another predictor of psychological traits; second, it renders intelligible the niche and rationality disconnect prevalent in the globalizing era.

## 1 Introduction

Strong Ties (ST) and Weak Ties (WT) refer to deep and narrow versus broad and shallow kinship ties, respectively. Cast in the framework of Curtin et al.’s (2020) concept of kinship intensity, ST societies are those with intensive kinship, “characterized by extended family networks, cousin marriage, polygyny, endogamy, unilineal descent, and an increase in relatedness within kin groups” (p. 417). WT societies are those with extensive kinship that “tend to encourage the formation of broad social networks with unrelated individuals, and often feature exogamy and bilateral descent” (p. 417). Consistent with the theory of ecological rationality (Todd and Gigerenzer, 2012) which posits that rationality and ecological niche have co-evolved, Sundararajan (2020) claims that ST and WT societies have fostered corresponding ST and WT rationalities.

### 1.1 Strong-ties and weak-ties rationalities

Rationality refers to reasoning about the world and the implicit logic behind such reasoning. According to Sundararajan (2020), strong-ties (ST) and weak-ties (WT) rationalities are kin-based reasoning—about our relatedness to others and to the world. ST rationality pertains to the orientation toward attunement to and preference for socially proximal others, such as kith and kin; WT rationality, the inclination and willingness to network with socially distal others, such as acquaintances and strangers. The theory of strong-ties and weak-ties rationalities has been applied to studies of ethnic minority groups in Asia (Sundararajan et al., 2022; Thong et al., 2023), predictions of COVID-related perceptions and behaviors across cultures (Ting et al., 2023; Zay Hta et al., 2024), decolonial psychology (Misra et al., 2024), and analysis of cultural loss in the globalizing era (Sundararajan et al., 2024). To understand the organizing and integrative function of these kin-based reasoning and logics in our lives, we turn to the theory of habitus by Bourdieu (1977).

### 1.2 Strong-ties and weak-ties rationalities as habitus

Habitus is defined by Bourdieu (1977) as socially inculcated “systems of durable, transposable dispositions” (p. 72). These enduring dispositions consist of a “socially constituted system of cognitive and motivating structures” (p. 76) which is what we call rationalities, as Bourdieu (1977) points out: “The habitus is the universalizing mediation which causes an individual agent’s practices, without either explicit reason or signifying intent, to be none the less ‘sensible’ and ‘reasonable’” (p. 79).

Corresponding to the ST and WT ecological niches are the two different economic cosmoses—“archaic” or “good-faith economy” (p. 172) in contrast to a market economy—noted by Bourdieu. The ST and WT rationalities behind these economic cosmoses are spelled out by Bourdieu (1977) as follows:

The general law of exchanges means that the closer the individuals or groups are in the genealogy, the easier it is to make agreements, the more frequent they are, and the more completely they are entrusted to good faith. Conversely, as the relationship becomes more impersonal, i.e., as one moves out from the relationship between brothers to that between virtual strangers (people from two different villages) or even complete strangers, so a transaction is less likely to occur at all, but it can become, and increasingly does become, purely “economic” in character, i.e., closer to its economic reality, and the interested calculation which is never absent even from the most generous exchange… can be more and more openly revealed. (p. 173)

There is growing evidence of the connection between variations in kinship intensity and cross-cultural differences in psychological traits (Enke, 2019; Henrich, 2020; Schulz et al., 2019; Talhelm et al., 2014), such as loyalty to in-group, trust toward strangers, individualism, differences in moral judgment, and so on. To explain this niche and trait connection, the theory of ST and WT rationalities postulates a cognitive and motivational system, habitus, as the underlying mechanism. More specifically, we claim that ST and WT rationalities are the mechanisms through which the alignment between ancestral niches and psychological traits is perpetuated through time. This claim is based on Bourdieu’s (1977) definition of habitus as:

…a subjective but not individual system of *internalized structures*, schemes of perception, conception, and action common to all members of the same group or class and constituting the precondition for all objectification and apperception” (p. 85, italics added).

Habitus not only internalizes the structure of the ecological niche, but also tends to “reproduce the objective structures of which they are the product” (p. 72). Thus, as habitus, ST and WT rationalities internalize and perpetuate the structure of the ancestral ecological niche, such as its preference for certain values, beliefs, and attitudes. This offers a plausible explanation for the impact of ancestral niches, such as rice versus wheat farming (Talhelm et al., 2014), on the psychological traits of contemporary populations. In the current study we test the hypothesis that ST (WT) rationalities tend to endorse the ST (WT) values and beliefs privileged by societies that have deep roots in the ST (WT) ancestral niches.

## 2 The current study

To test the hypothesis that ST and WT rationalities, when activated, will endorse corresponding ST and WT values and beliefs, respectively, we conducted a large-scale study across four societies—China, India, Taiwan, and the United States. In cross-cultural studies, China and the United States have served as representative samples of collectivism and individualism, respectively. Improving on this convention, we added the ideal type approach in which samples represent not populations so much as theoretically relevant categories. Thus, we compare Han Chinese with White Americans as representative samples of societies with ST versus WT ancestral niches, respectively, and added Taiwan and India to explore variations within ST societies. We expected the ST protocols to vary between India and China due to their differences in ancestral niches. Although China and Taiwan differ in their political structures, these two societies may not differ in rationality, since they share the same ancestral niche.

### 2.1 Research design

We first administered the *Strong-Ties Weak-Ties Rationality Scale (STWTRS)* to activate ST and WT reasoning and logic. Subsequently, the participants were asked to rate a list of values and beliefs that had well known connections with ST or WT ancestral niches. More specifically, the following values and beliefs are referred to as ST values and beliefs, due to their documented (Enke, 2019; Schulz et al., 2019) prevalence in ST societies: loyalty to family and ingroup, deference to authority, concern with purity, and belief in a just world (BJW) (Wu et al., 2011). To assess these ST values and beliefs, we selected measurements from previous studies (Sundararajan and Yeh, 2022; Yeh et al., 2022) that showed positive associations with ST rationality: *Reciprocal Filial Piety, Authoritarian Filial Piety, Ingroup bias, (deference to) Authority, Purity,* and *Belief in a Just World*. As explained in the Measures section, we dropped the *Ingroup bias* scale due to its low reliability and combined *Authority* and *Purity* into one scale, here called *Dharma*.

As Curtin et al. (2020) point out, people in “societies with loose kinship” (p. 419) tend to be “individualistic, independent, self-oriented, and concerned about creativity” (p. 418); and in their moral reasoning to “place more emphasis on universal moral values” (p. 419). We refer to these individualistic and universalizing values and beliefs as WT values and beliefs. For the measurement of WT moral values and beliefs, we intended to use the following scales that in previous studies (Sundararajan and Yeh, 2022; Yeh et al., 2022) showed positive associations with WT rationality: *Care, Fairness*, and *Belief in an Unjust World*. But, as explained in the Measures section below, *Care* and *Fairness* scales had to be dropped due to low reliability, leaving *Belief in an Unjust World* as the sole measure of WT values and beliefs.

### 2.2 Hypothesis testing

Based on Bourdieu’s (1977) formulation of habitus, “As an acquired system of generative schemes objectively adjusted to the particular conditions in which it is constituted, the habitus engenders all the thoughts, all the perceptions, and all the actions consistent with those conditions, *and no others*” (p. 95, italics added), we made the following predictions: ST and WT rationalities, being the internalization of the social structures of ST and WT ancestral niches which privileged ST and WT values and beliefs, will reproduce the same structure by endorsing ST and WT values and beliefs, respectively. Thus,

*Hypothesis 1 (H1)*: ST rationality will endorse ST values and beliefs and WT rationality will endorse WT values and beliefs. Specifically,

*H1a*: ST rationality will be positively related to **Reciprocal Filial Piety**.

*H1b*: ST rationality will be positively related to **Authoritarian Filial Piety**.

*H1c*: ST rationality will be positively related to **Dharma (Purity + Authority)**.

*H1d*: ST rationality will be positively related to **Belief in a Just World**.

*H1e*: WT rationality will be positively related to **Belief in an Unjust World**.

Next, we test societal moderating effects, with a special focus on the ancestral niche. The connection between (ST and WT) rationalities and their associated ancestral niches is underscored by Bourdieu’s (1977) claim that habitus as “systems of durable, transposable dispositions” (p. 72) is “history turned into nature” (p. 78). More specifically, he states that “the habitus is an endless capacity to engender products – thoughts, perceptions, expressions, actions—whose limits are set by the historically and socially situated conditions of its production” (p. 95).

To classify societies based on their ancestral niches, we consider Asian societies, such as China, India, and Taiwan, as ST societies due to their long-standing agriculture-based ecologies; we consider Americans with European ancestry a sample of WT society due to their roots in Medieval Christianity (Schulz et al., 2019) that witnessed “the broad weakening of kinship as a central organizing force” (Curtin et al., 2020, p. 417). Drawing on this ancestral-niche perspective, we proposed that the effects of ST and WT rationalities in H1 will be amplified in ST or WT societies, respectively. We predict that:

*Hypothesis 2 (H2)*: The effects of ST and WT rationalities will be stronger in societies with ST or WT ancestral niches, respectively. Specifically,

*H2a*: The relationship between ST rationality and **Reciprocal Filial Piety** will be stronger in a ST society than that in a WT society.

*H2b*: The relationship between ST rationality and **Authoritarian Filial Piety** will be stronger in a ST society than that in a WT society.

*H2c*: The relationship between ST rationality and **Dharma (Purity + Authority)** will be stronger in a ST society than that in a WT society.

*H2d*: The relationship between ST rationality and **Belief in a Just World** will be stronger in a ST society than that in a WT society.

*H2e*: The relationship between WT rationality and **Belief in an Unjust World** will be stronger in a WT society than that in a ST society.

Among Asian ST societies, we expect regional differences. Since Mainland Chinese and Taiwanese share the same ancestral niche, we did not expect regional difference in rationality between the two and grouped them as Chinese societies. Judging by the percentage of atheists in their society, (India, 1.9%; China, 71%; Taiwan, 37%), Indians (84% of whom identified themselves as Hindus) are evidently more religious than their Chinese counterparts. According to Saroglou et al. (2004), religiosity and religion are found to be associated with the retention of traditional beliefs and practices. Thus, Indian society is likely to have preserved its ST traditions more than Chinese societies. We therefore hypothesize:

*Hypothesis 3 (H3)*: The effects of ST rationality will be different among the ST societies, i.e., India and Chinese societies. Specifically,

*H3a*: The relationship between ST rationality and **Reciprocal Filial Piety** will be stronger in India than that in Chinese societies.

*H3b*: The relationship between ST rationality and **Authoritarian Filial Piety** will be stronger in India than that in Chinese societies.

*H3c*: The relationship between ST rationality and **Dharma (Purity + Authority)** will be stronger in India than that in Chinese societies.

*H3d*: The relationship between ST rationality and **Belief in a Just World** will be stronger in India than that in Chinese societies.

## 3 Method

### 3.1 Sample and procedures

This study was administered as an online survey of opinions concerning the COVID-19 pandemic. Participants provided their informed consent after reading the survey statement that included the purpose of study, eligibility for participation, required time, compensation, and the anonymity of their responses. We recorded a total of 1,291 valid participants across four societies: China (*n* = 359), India (*n* = 264), Taiwan (*n* = 289), and the United States (*n* = 379), with the last group comprising only non-Asian Americans with primarily European ancestry. The recruitment of participants received ethics clearance through the IRB (institutional review board) of the first author. Detailed recruitment procedures are provided in Supplementary Appendix 1.

The demographic information for each society is presented in Table 1. Overall, the sample had slightly more females (57.24%) than males, with an average age of 34.80 (SD = 12.04). Participants were highly educated, with 90% holding an associate degree or above. Additionally, they are from a spectrum of socio-economic classes, about half ranking themselves from the middle class.

**Table 1:** Demographics by society.

Society	*N*	Age		Education		Socio-economic status, SES		Gender (% female)	% living with extended family
		Mean	S.D.	Mean	S.D.	Mean	S.D.		
China	359	31.46	(8.48)	4.00	(0.69)	5.28	(1.55)	50.97	55.15
India	264	29.75	(12.00)	5.08	(1.14)	6.34	(1.80)	71.21	82.58
Taiwan	289	33.84	(9.63)	5.02	(0.81)	4.95	(1.55)	58.48	65.40
U.S.	379	42.23	(13.08)	4.66	(0.91)	6.12	(1.65)	52.50	22.69
All	1,291	34.80	(12.04)	4.64	(0.99)	5.67	(1.72)	57.24	53.52

S.D. = standard deviation; age in years; education attainment (1 = elementary school; 2 = junior high school; 3 = senior high school; 4 = Associate Degree; 5 = College Degree; 6 = Graduate school and above); SES (“1” lowest to “10” highest); gender (binary options: male and female); living with extended family (Yes or No).

### 3.2 Measures

Unless stated otherwise, we adopted a six-point Likert scale (1-completely/2-strongly/3-slightly disagree; 4-slightly/5-strongly/6-completely agree) for measuring all study variables. All measures are bilingual (for details see Yeh et al., 2022)—an English version was used for the US and India samples, and a Chinese version, for the China and Taiwan samples.

#### 3.2.1 Strong-ties and weak-ties rationality scale

The STWTRS was developed to activate ST and WT rationalities (Sundararajan and Yeh, 2022). Given that COVID-19 has become a global experience since 2020, the pandemic scenario was used as a context to anchor STWTRS construction. The 20 items of the STWTRS are evaluative statements concerning COVID-related precepts, propositions, and practices, which were deemed congruent with the logic of ST or WT rationalities (for details, see Sundararajan and Yeh, 2022; Yeh et al., 2022). A sample item of ST rationality is, “It is important not to complain or gripe during a lockdown because these words spread negative energy and can hurt many people around you.” The logic behind this item pertains to a group-orientedness characteristic of ST rationality. A sample item of WT rationality is, “In implementing a lockdown, it is important to balance the interests of the group with the interests of individuals so that one is not served at the expense of the other.” The logic behind this item pertains to the analytical thinking characteristic of WT rationality. The compatibility between the respective items and the ST and WT rationalities was supported by factor analyses presented in the studies of Yeh et al. (2022) and Ting et al. (2023), which showed that STWTRS items loaded in their predicted directions.

#### 3.2.2 Dual filial piety

We measured dual filial piety with the 16-item scale developed by Yeh and Bedford (2003). This scale measures peoples’ opinions about the way they treat their parents. We asked respondents to rate the importance of each statement on a six-point scale. The rating scale used extremely unimportant (coded “1”) and extremely important (coded “6”) as the anchors. The scale has two aspects, reciprocal filial piety (RFP) and authoritarian filial piety (AFP), each comprising eight items. A sample item of RPF is, “Be grateful to my parents for raising me.” A sample item of APF is, “Do whatever my parents ask right away.” The scale and its two aspects have been supported with good reliability and validity in previous studies (Wu and Yeh, 2021; Lim et al., 2022).

#### 3.2.3 Belief in a just world and belief in an unjust world

We utilized Dalbert et al. (2001)’s 10-item scale to gage participants’ perceptions of the fairness of the world. In our analysis, we divided the scale into two subscales: the first six, positively worded items measured just-world beliefs, while the last four, negatively worded items assessed unjust-world beliefs (BUJW). A sample item of BJW is, “I think basically the world is a just place.” A sample item of BUJW is, “A lot of people suffer an unjust fate.” We made this division for two reasons: first, the fit indices in the four single-society confirmatory factor analyses (CFAs) on a one-factor model were not acceptable, and second, subsequent single-society exploratory factor analyses suggested that the data clustered into two distinct factors in the three Asian societies with good fit and in the US with marginally acceptable fit, thereby justifying the split of the original scale into two factors.

#### 3.2.4 Dharma

We combined the three-item authority sub-scale and the three-item purity sub-scale of Moral Foundations Evaluation from the Moral Foundations Questionnaire (MFQ) (Graham et al., 2011) to form the variable we labeled as “Dharma.” Dharma refers to the basic norms in the world that humans have the obligation to follow. Authority and purity are two such basic norms—the former pertains to the hierarchical order of society that we need to honor (defer to those in authority), while the latter pertains to the basic norms of human nature (such as the incest taboo), violation of which degrades our being as humans. We did not use the other three MFQ sub-scales because of their low reliabilities across these four societies as measured by Cronbach’s alpha (care: range of 0.30–0.60; fairness: range of 0.31–0.49; ingroup: range of 0.37–0.60). The composite Dharma scale of authority and purity yielded acceptable alphas across our four samples (ranging from 0.67 to 0.83).

#### 3.2.5 Covariates

We controlled for the demographic variables that the STWTRS was found to be related to Yeh et al. (2022), i.e., age (years), gender (male/female), education attainment (“1” elementary school; “6” graduate school or above), socio-economic status (“1” the lowest to “10” the highest), and living with extended family (Yes/No).

### 3.3 Measurement model

#### 3.3.1 Measurement invariance

Given that participants came from four different societies, there could be variations in how they interpreted the scale structure (configurable variance), as well as in their use of measurement units (metric variance and scalar variance) (Van de Vijver and Leung, 2011). To address this issue, we assessed the measurement invariance of each of the seven (sub)scales using the approximate alignment method with maximum likelihood estimation (Muthén and Asparouhov, 2014).

Based on the alignment results, we removed the items that had either non-invariant loadings or intercepts in two or more societies. As a result, we removed four items from ST rationality, one item each from *Reciprocal Filial Piety; Authoritarian Filial Piety; Dharma (Purity + Authority); and Belief in a Just World*, but none from WT rationality and *Belief in an Unjust World*. All resulting non-invariance rates (ranging from 3.13 to 12.50%) were less than the cut-off criterion of 25% (Muthén and Asparouhov, 2014, pp. 3–4). We can conclude that we have secured trustworthy alignment results for these seven, unifactorial scales. The specific non-invariance rates for intercepts and loadings for the scales are shown in Supplementary Appendix 2.

#### 3.3.2 Confirmatory factor analysis

We conducted a confirmatory factor analysis for the entire sample to validate our measurement model. The seven-factor model exhibited an acceptable fit (*N* = 1,291, *χ*^2^_(594)_ = 2199.634, CFI = 0.917, TLI = 0.901, RMSEA = 0.046, SRMR = 0.060) after we removed those items that had a factor loading below 0.40. That procedure resulted in further removal of five items from the STWTR scale. Supplementary Appendix 3 provided the final scales used in this study.

To ensure the discriminant and convergent validity of the factors in the measurement model, we combined the factors and formed three alternative models: one with four factors [ST and WT rationality scales; Reciprocal Filial Piety and Authoritarian Filial Piety; Belief in a Just World, and Belief in an Unjust World; and Dharma (Purity + Authority); one with three factors (rationality scales plus filial piety scale; Belief in a Just World and Belief in an Unjust World; and Dharma), and one with only one factor (all items)]. All alternative models (Table 2) exhibited a poorer fit; hence, we concluded that the seven-factor measurement model demonstrated acceptable discriminant and divergent properties.

**Table 2:** Confirmatory factor analysis of alternative measurement models.

Model	χ^2^	df	RMSEA*	CFI	TLI	SRMR
7-factor (STR, WTR, RFP, AFP; BJW, BUJW, Dharma)	2199.634	594	0.046 (0.044–0.048)	0.917	0.901	0.060
4-factor (STR + WTR; RFP + AFP; BJW + BUJW; Dharma)	3920.00	609	0.065 (0.063–0.067)	0.828	0.802	0.086
3-factor (STR + WTR + RFP + AFP; BJW + BUJW; Dharma)	4623.861	612	0.071 (0.067–0.073)	0.792	0.761	0.087
1-factor (all scales combined)	5422.83	615	0.078 (0.076–0.080)	0.751	0.715	0.092

AFP, authoritarian filial piety; BJW, belief in a just world; BUJW, belief in an unjust world; RFP, reciprocal filial piety; STR, strong-ties rationality; WTR, weak-ties rationality; df, degree of freedom; RMSEA, root mean square error of approximation; CFI, comparative fit index; TLI, Tucker-Lewis index; SRMR, Standardized root mean square residual; *90% confidence interval values in brackets.

We computed the society means, standard deviations, and Cronbach’s alphas for all study variables (see Table 3). The Cronbach’s alpha of the total sample for ST rationality was 0.73 (range of 0.60–0.79 in the four societies); for WT rationality, was 0.61 (range of 0.55–0.76); for Reciprocal Filial Piety, was 0.87 (range of 0.81–0.92); for Authoritarian Filial Piety, was 0.86 (range of 0.79–0.87); for Belief in a Just World was 0.84 (range of 0.74–0.88); for Belief in an Unjust World, was 0.81 (range of 0.67–0.91); and lastly for Dharma (Purity + Authority), was 0.76 (range of 0.60–0.79).

**Table 3:** Means, standard deviations and Cronbach’s alphas of the study variables by society.

	China	India	Taiwan	U.S.
Strong-ties rationality				
Mean (S.D.)	4.91 (0.72)	4.87 (0.65)	4.67 (0.76)	4.25 (0.82)
Alpha	0.73	0.60	0.79	0.70
Weak-ties rationality				
Mean (S.D.)	4.71 (0.59)	5.05 (0.55)	4.87 (0.61)	4.71 (0.77)
Alpha	0.55	0.55	0.76	0.71
Reciprocal filial piety				
Mean (S.D.)	5.32 (0.53)	5.60 (0.50)	5.06 (0.69)	5.04 (0.81)
Alpha	0.81	0.85	0.92	0.87
Authoritarian filial piety				
Mean (S.D.)	3.02 (0.74)	3.82 (1.06)	3.15 (0.92)	2.41 (0.85)
Alpha	0.79	0.84	0.87	0.82
Belief in a just world				
Mean (S.D.)	4.11 (0.81)	4.12 (0.94)	3.64 (0.86)	3.30 (1.08)
Alpha	0.74	0.77	0.82	0.88
Belief in an unjust world				
Mean (S.D.)	2.99 (0.93)	2.69 (0.82)	2.52 (0.90)	2.83 (0.98)
Alpha	0.79	0.67	0.91	0.84
Dharma				
Mean (S.D.)	4.49 (0.75)	4.26 (0.92)	4.27 (0.84)	3.50 (1.15)
Alpha	0.76	0.60	0.68	0.79

Standard deviations in brackets.

All alphas met the acceptable range (>0.60) for cross-cultural research (Fu and Yukl, 2000), except for the WT rationality scales of China and India (=0.55). In the related main effect analyses using WT rationality as a predictor, the coefficient directions were consistent in each model with or without China/India and were statistically significant except in the relationship with Belief in an Unjust World where the *p-*value (without China) was 0.054 indicating a marginally acceptable finding. We therefore retained China and India in the related analyses.

### 3.4 Analytic strategy

Given the nested structure of our data (individuals within societies), the ideal approach would be to use multilevel modeling to test our hypotheses. However, with only four societies in our dataset, we could not employ multilevel modeling, as it requires at least 10 societal-level clusters (Hox et al., 2010). Consequently, we opted to use linear regressions.

First, to assess the main effects, we regressed the five belief and value outcome variables on the predictors, ST and WT rationality, and the covariates. Next, to discern any society (ecological niche)-based moderating effects, we employed a systematic “zooming-in” strategy. This involved introducing distinct dummy variables one by one, which include “strong-ties society vs. non-strong-ties society,” “India vs. non-India,” and “China vs. non-China (i.e., Taiwan).”

The “strong-ties vs. non-strong-ties society” comparison highlighted a typical non-WEIRD vs. WEIRD (Western, Educated, Industrialized, Rich and Democratic) disparity, while the “India vs. non-India (China and Taiwan)” contrast underscored a regional (Asia)-cluster effect. Though we did not hypothesize a China vs. Taiwan contrast rooted in the ancestral niche perspective, we examined it for the sake of comprehensiveness in reporting the results of our analyses.

Our procedure began by assessing a moderating effect from strong-ties societies. This was done to evaluate how the main effect in question varies in strength across different societal contexts. To ascertain if such an effect existed, we excluded the US data (our sole weak-ties society) and focused on the Asian subset (*N* = 912) to ascertain if there was any moderating effect resulting from India’s societal context. If confirmed, we then excluded the Indian data, narrowing our focus to the Chinese subset (*N* = 648), where we used the China vs. Taiwan dummy to check for plausible moderating effects resulting from the societal contexts of China and Taiwan. We applied this systematic approach for all outcomes, halting the process if any stage yielded non-significant results. Through this pseudo-multilevel methodology (as we label it), we could probe the macro-cultural divides on global (strong-ties vs. weak-ties societies), regional (India vs. East Asia), and sub-regional (China vs. Taiwan) scales.

## 4 Results

The means, standard deviations, and zero-order correlation coefficients of this study’s main variables are provided in Table 4. Both ST and WT rationalities were positively correlated with ST society (ST rationality: *r* = 0.33, *p* < 0.001; WT rationality: *r* = 0.10, *p* < 0.001). But while WT rationality was positively correlated with socio-economic status (*r* = 0.10*, p < 0.*001), ST rationality was negatively correlated with education (*r* = −0.10*, p < 0.*001), suggesting that the participants in our sample with a WT orientation were more affluent and educated, while those with ST orientation tended to have lower levels of education.

**Table 4:** Means, standard deviations, and zero-order correlation coefficients.

	Mean	S.D.	1	2	3	4	5	6	7	8	9	10	11	12
1. ST rationality	4.65	0.80												
2. WT rationality	4.81	0.66	0.54***											
3. RFP	5.24	0.69	0.42***	0.40***										
4. AFP	3.03	1.01	0.40***	0.20***	0.37***									
5. BJW	3.77	1.00	0.41***	0.17***	0.27***	0.46***								
6. BUJW	2.78	0.93	−0.07*	0.04***	−0.04	−0.08**	−0.44***							
7. Dharma	4.10	1.02	0.51***	0.25***	0.36***	0.56***	0.54***	−0.19***						
8. Age	34.81	12.04	−0.03	0.05	−0.08**	−0.03	−0.05	−0.15***	0.02					
9. Gender	1.57	0.49	0.04	0.01	0.10**	−0.10***	0.02	−0.01	−0.04	−0.11***				
10. Education	4.64	0.99	−0.10***	0.03	−0.01	0.06*	−0.06*	0.01	−0.12***	0.046	0.02			
11. SES	5.67	1.72	0.03	0.10***	0.02	0.03	0.03	−0.07*	−0.05	0.08**	−0.02	−0.04		
12. Extended fam	0.54	0.50	0.14***	0.06*	0.16***	0.23***	0.11***	0.14***	0.11***	−0.43***	0.10**	−0.01	−0.07*	
13. ST society	0.71	0.46	0.33***	0.10***	0.19***	0.39***	0.30***	0.04	0.38***	−0.40***	0.06*	−0.01	−0.17***	0.40***

*p < 0.05; **p < 0.01; ***p < 0.001. ST, strong-ties; WT, weak-ties; RFP, reciprocal filial piety; AFP, authoritarian filial piety; BJW, belief in a just world; BUJW, belief in an unjust world; age coded in years; gender (1 = male; 2 = female); education (1 for elementary school, 6 = graduate school or above); SES (“1” the lowest to “10” the highest); extended fam = extended family (1 = yes, 0 = no); ST society coded “1 = Yes, 0 = No”.

### 4.1 Main effects

Tables 5–7 provide the regression results. Table 8 presents the summary of the findings of all hypothesis testing. Table 9 highlights the directions of the significant main and moderating effects. H1 stated that ST rationality would be positively related to ST values and beliefs of reciprocal filial piety, authoritarian filial piety, Dharma (purity + authority), and belief in a just world, and that WT rationality would be positively related to the WT values and belief in an unjust world. Regression results in Models 1a to 1e (Table 5) showed that controlling for age, gender, educational attainment, social economic status, and living-with-extended-family status, ST rationality was positively related to reciprocal filial piety (*β* = 0.24, *p* < 0.001), authoritarian filial piety (*β* = 0.52, *p <* 0.001), Dharma [purity + authority (*β* = 0.65, *p* < 0.001)], belief in a just world (*β* = 0.55, *p* < 0.001), and WT rationality was positively related to belief in an unjust world (*β* = 0.68, *p* < 0.001), hence supporting hypotheses H1a to H1e.

**Table 5:** Main effects of strong-ties rationality and weak-ties rationality on values and beliefs.

	Model 1a		Model 1b		Model 1c		Model 1d		Model 1e	
Dependent variable	Reciprocal filial piety		Authoritarian filial piety		Dharma		Belief in a just world		Belief in an unjust world	
	Coeff.	s.e.	Coeff.	s.e.	Coeff.	s.e.	Coeff.	s.e.	Coeff.	s.e.
Intercept	2.714***	0.176	0.363	0.260	1.730***	0.253	1.740***	0.264	18.675***	1.059
Age	−0.002	0.002	0.004	0.002	0.006*	0.002	−0.001	0.002	−0.034***	0.009
Gender	0.100**	0.034	−0.274***	0.051	−0.114*	0.049	−0.006	0.052	−0.203	0.207
Education	0.011	0.017	0.109***	0.026	−0.072**	0.025	−0.014	0.026	−0.007	0.104
SES	−0.002	0.010	0.021	0.015	−0.040**	0.014	0.015	0.015	−0.138*	0.059
Extended family	0.120**	0.038	0.421***	0.056	0.150**	0.054	0.103	0.057	0.794***	0.227
ST rationality	0.237***	0.026	0.515***	0.038	0.652***	0.037	0.550***	0.039	−0.677***	0.154
WT rationality	0.256***	0.031	−0.059	0.046	−0.042	0.044	−0.116*	0.046	0.680***	0.185
*N*	1,291		1,291		1,291		1,291		1,291	
*F*	56.37***		51.95***		69.86***		38.63***		9.69***	
Adjusted *R*^2^	0.231		0.217		0.272		0.170		0.045	

*p < 0.05; **p < 0.01; ***p < 0.001; Extended family (coded “1” for Yes, “0” for No); Gender: 1 = male; 2 = female; SES socio-economic status (coded “1” lowest, to “10” highest); ST Rationality: strong-ties rationality; WT Rationality: weak-ties rationality.

**Table 6:** Interaction of strong-ties (weak-ties) rationality and strong-ties (weak-ties) society.

	Model 2a		Model 2b		Model 2c		Model 2d		Model 2e	
Dependent variable	Reciprocal filial piety		Authoritarian filial piety		Dharma		Belief in a just world		Belief in an unjust world	
	Coeff.	s.e.	Coeff.	s.e.	Coeff.	s.e.	Coeff.	s.e.	Coeff.	s.e.
Intercept	2.937***	0.215	0.537	0.303	1.966***	0.294	2.262***	0.315	17.424***	1.412
Age	−0.002	0.002	0.012***	0.002	0.013***	0.002	0.004	0.002	−0.037***	0.010
Gender	0.107**	0.035	−0.257***	0.049	−0.096*	0.047	0.017	0.050	−0.207	0.206
Education	0.011	0.017	0.101***	0.024	−0.080**	0.024	−0.017	0.025	0.009	0.104
SES	−0.002	0.010	0.042**	0.014	−0.020	0.014	0.026	0.015	−0.143*	0.060
Extended family	0.112**	0.039	0.276***	0.055	−0.011	0.053	0.010	0.057	0.828***	0.234
ST rationality	0.166***	0.041	0.253***	0.058	0.384***	0.057	0.273***	0.061	−0.623***	0.163
WT rationality	0.261***	0.031	−0.019	0.044	−0.002	0.042	−0.084	0.045	0.936***	0.261
ST society	−0.376	0.216	−0.138	0.304	−0.251	0.296	−0.797*	0.317	1.966	1.533
ST rationality*ST society	0.094*	0.047	0.190**	0.067	0.209**	0.065	0.283***	0.069		
WT rationality*WT society									0.462	0.316
										
*N*	1,291		1,291		1,291		1,291		1,291	
*F*	44.48***		59.47***		74.68***		39.02***		7.87***	
Adjusted *R*^2^	0.233		0.290		0.340		0.210		0.046	

*p < 0.05; **p < 0.01; ***p < 0.001; ST society = Strong-ties society (coded “1” for Yes; “0” for No); WT society = Weak-ties society (coded “1” for Yes; “0” for No).

**Table 7:** Interaction of strong-ties rationality and India (or China).

	Model 3a		Model 3b		Model 3c		Model 3d		Model 3e	
Dependent variable	Reciprocal filial piety		Authoritarian filial piety		Dharma		Belief in a just world		Authoritarian filial piety	
	Coeff.	s.e.	Coeff.	s.e.	Coeff.	s.e.	Coeff.	s.e.	Coeff.	s.e.
Intercept	2.910***	0.203	1.132***	0.321	1.198***	0.275	1.503***	0.312	0.965*	0.444
Age	−0.007***	0.002	0.019***	0.003	0.010***	0.003	−0.001	0.003	0.017***	0.004
Gender	0.061	0.035	−0.318***	0.056	−0.183***	0.048	0.002	0.055	−0.280***	0.058
Education	−0.024	0.018	0.056	0.028	−0.033	0.024	−0.041	0.028	−0.025	0.042
SES	−0.007	0.011	−0.008	0.017	−0.014	0.014	0.070***	0.016	0.051**	0.019
Extended family	0.039	0.039	0.189**	0.062	0.060	0.053	0.018	0.062	0.162*	0.063
ST rationality	0.248***	0.032	0.377***	0.051	0.541***	0.043	0.506***	0.050	0.426***	0.068
WT rationality	0.282***	0.034	−0.067	0.054	0.155**	0.047	−0.017	0.423	−0.035	0.060
India/China^#^	0.350	0.270	−0.454	0.427	−0.014	0.366	0.053	0.423	0.463	0.386
ST rationality*India	−0.013	0.055	0.250**	0.087	−0.024	0.075	0.015	0.086		
ST rationality*China									−0.144	0.079
*N*	912		912		912		912		648	
*F*	48.75***		45.27***		50.71***		27.62***		19.48***	
Adjusted *R*^2^	0.321		0.304		0.329		0.208		0.216	

*p < 0.05; **p < 0.01; ***p < 0.001. ^#^ India, coded “1” and non-India [China or Taiwan] coded “0,” is a moderator for Model 3a-3d. China, coded “1” and Taiwan coded “0,” is a moderator for Model 3e.

**Table 8:** Summary of findings of hypothesis testing.

Hypothesis	Description	Result
H1a	ST rationality is positively related to reciprocal filial piety (RFP)	Supported
H1b	ST rationality is positively related to authoritarian filial piety (AFP)	Supported
H1c	ST rationality is positively related to Dharma	Supported
H1d	ST rationality is positively related to belief in a just world (BJW)	Supported
H1e	WT rationality is positively related to belief in an unjust world (BUJW)	Supported
H2a	The relationship between strong-ties rationality and RFP will be stronger in a strong-ties society than that in a weak-ties society	Supported
H2b	The relationship between strong-ties rationality and AFP will be stronger in a strong-ties society than that in a weak-ties society	Supported
H2c	The relationship between strong-ties rationality and Dharma will be stronger in a strong-ties society than that in a weak-ties society	Supported
H2d	The relationship between strong-ties rationality and BJW will be stronger in a strong-ties society than that in a weak-ties society	Supported
H2e	The relationship between weak-ties rationality and BUJW will be stronger in a weak-ties society than that in a strong-ties society	Not supported
H3a	The relationship between strong-ties rationality and RFP will be stronger in India than that in Chinese societies	Not supported
H3b	The relationship between strong-ties rationality and AFP will be stronger in India than that in Chinese societies	Supported
H3c	The relationship between strong-ties rationality and Dharma will be stronger in India than that in Chinese societies	Not supported
H3d	The relationship between strong-ties rationality and BJW will be stronger in India than that in Chinese societies	Not supported

**Table 9:** Significant main effects and moderating effects.

Relationship	Main effect	ST society vs non-ST society	India vs Non-India (i.e., Chinese societies)	China vs Taiwan
ST rationality-RFP	Yes	Yes	No	/
ST rationality-AFP	Yes	Yes	Yes	No
ST rationality-Dharma	Yes	Yes	No	/
ST rationality-BJW	Yes	Yes	No	/
WT rationality-BUJW	Yes	No	/	/

ST, strong-ties; WT, weak-ties; RFP, reciprocal filial piety; AFP, authoritarian filial piety; BJW, belief in a just world; BUJW, belief in an unjust world. All significant effects were positive unless stated otherwise.

### 4.2 Society moderating effects

**Figure 1:**
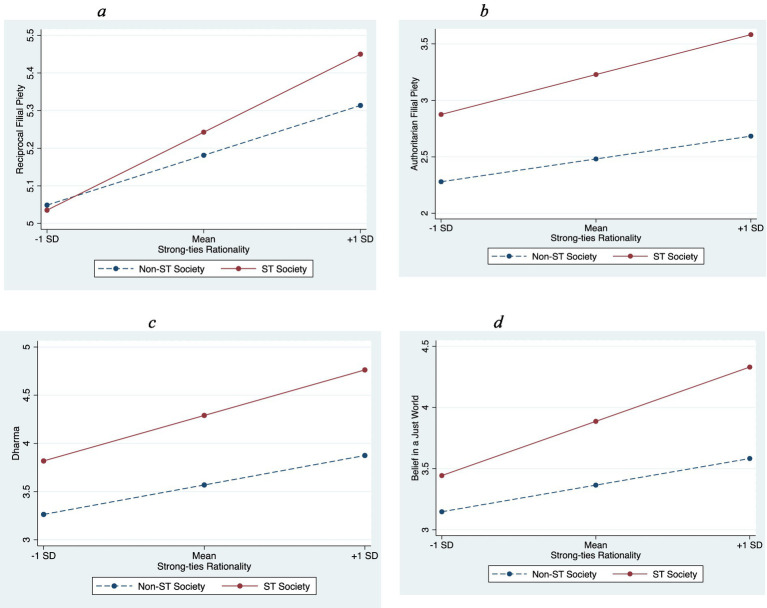
Significant interaction of strong-ties rationality and strong-ties (ST) society. (A) Reciprocal filial piety. (B) Authoritarian filial piety. (C) Dharma. (D) Belief in a just world.

H2 and H3 stated that there would be moderating effects by ST vs. WT societies, and regional-cluster moderating (India vs. China and Taiwan) effects, respectively, on the matching ST (WT) rationality and ST (WT) values and beliefs relationships. As hypothesized (presented in Models 2a-2d in Table 6 and Figures 1A–D), strong-ties society strengthened the positive relationship of ST rationality with reciprocal filial piety (*β* = 0.09, *p* < 0.05), with authoritarian filial piety (*β* = 0.19, *p* < 0.01), with Dharma (purity + authority) (*β* = 0.21, *p* < 0.01), and with belief in a just world (*β* = 0.28, *p* < 0.001). Model 2e shows that weak-ties society did not moderate the WT rationality- belief in an unjust world relationship (*β* = 0.46, ns). Therefore, H2a, H2b, H2c, and H2d were supported, but H2e was not. See Figures 1A–D for the moderation effect plots.

**Figure 2:**
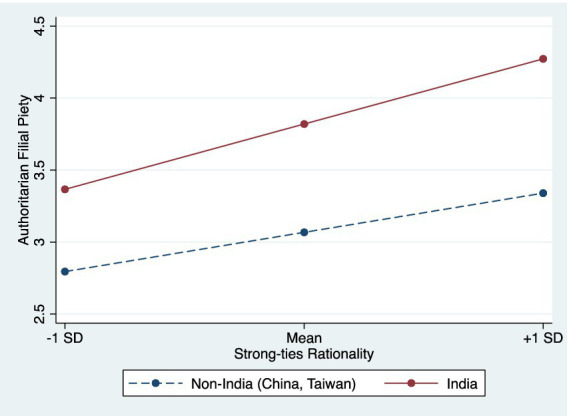
Significant interaction between strong-ties rationality and India.

Given these results, we proceeded with the pseudo-multilevel strategy, and opted to exclude the U.S. data and examined H3a to H3d. We used the Asian data to test if the strengths of the relationships between ST rationality and ST values and beliefs (reciprocal filial piety, authoritarian filial piety, Dharma, and belief in a just world) varied between India and the Chinese societies. As shown in 7 and Figure 2, India demonstrated a magnifying effect on the ST rationality-authoritarian filial piety relationship (*β* = 0.25, *p* < 0.01; Model 3b). No significant effect was observed on ST rationality’s relationship with reciprocal filial piety (Model 3a), Dharma (Model 3c), or belief in a just world (Model 3d). Hence, only H3b was supported, while H3a, H3c and H3d were not. Then, we tested if the relationship between ST rationality and authoritarian filial piety varied between China and Taiwan. The result showed that the interaction term was not significant, indicating the strength of the relationships remained consistent across the two Chinese societies (Model 3e).

In conclusion, as summarized in Table 8, H1 was fully supported; H2 was mostly supported except for one non-significant societal moderating effect on the relationship between WT rationality and belief in an unjust world; and H3 was partially supported with one significant societal moderating effect on the relationship between ST rationality and authoritarian filial piety.

## 5 Discussion

The results for the main effects (see Table 5) of ST and WT rationalities are as predicted. There were consistent significant and positive associations between ST rationality and ST values and beliefs—*Reciprocal Filial Piety; Authoritarian Filial Piety; Dharma (Purity + Authority); Belief in a Just World* and between WT rationality and WT value and belief (*Belief in an Unjust World*).

The interaction effect of ST vs. WT society and rationalities suggests that ST society amplified all the relationships between ST rationality and ST values and beliefs. This claim is not conclusive, as we did not observe the same amplifying effect in the link between WT rationality and its associated value and belief (*Belief in an Unjust World*). The absence of a societal moderating effect could mean the relationship between WT rationality and *Belief in an Unjust World* is universal, at least it was across our sub-samples. Across both ST and WT societies, WT thinking leads to the consistent belief in an unjust world. Another possible explanation for the universal endorsement (by WT rationality) of *Belief in an Unjust World* is globalization which has contributed to the spread of WT values and beliefs (Welzel and Inglehart, 2010). Measurement of more values and beliefs associated with WT rationality across more societies is needed to test this plausibility.

Within Asian (ST) societies, we observed one moderating effect: the relationship between ST rationality and *Authoritarian Filial Piety* (AFP) was stronger in India. There are two possible and mutually reinforcing explanations that can be derived from the study of Yeh et al. (2013): First, the practice of AFP has declined in Chinese societies. Second, AFP was found to be associated with observance of conservative traditional norms, which supports our prediction that India is more conservative than Chinese societies. In conclusion, our approach of zooming in to unpack the moderating effects of society revealed a global effect along the “strong-ties versus weak-ties” societal divide, with minimal cultural differences across regions. This is consistent with the protocol of cultural contrasts in which differences within ST societies are smaller than differences between ST and WT societies.

Lastly, our analytical strategy for unpacking the societal moderating effects on individual-level relationships is novel. The conventional approach, which involves analyzing society-by-society comparisons using society averages, has been criticized as limited by cross-cultural researchers, such as Ralston et al. (2023) and Smith and Bond (2019). Our pseudo-multilevel approach to analyzing small multi-country datasets has yielded intriguing results while preserving the integrity of individual-level findings. Although it is still a rudimentary way to analyze cross-society data, it is superior to using society averages and the traditional group comparison method.

### 5.1 Potential contributions to cultural and cross-cultural psychology

Our proposed mental model of kinship intensity not only explains the well documented connection between niche and psychological traits, but also makes potential contributions to cultural and cross-cultural psychology. In conventional research on collectivism and individualism, culture refers to both societies as well as psychological traits. One major contribution of the kinship intensity research lies in opening the black box of culture by measuring society specifically with a kinship intensity index (Schulz et al., 2019; Curtin et al., 2020) that covers history, social institutions, and many other social-ecological factors. Using this social-ecological index to predict psychological traits, the kinship intensity paradigm has created a two-component model of culture. Opening the black box of culture further, we have offered a three-component model of culture: mind (rationality), ecological niche, and cultural-psychological traits.

Our three-component model of culture is consistent with the complementarity theory of Fiske (2000), who claims that “The key concept in complementarity theory is that people have highly structured, evolved (predominately universal), attentional, motivational, cognitive, and developmental *proclivities* for discerning congruent cultural paradigms and using them to construct and utilize local cultural coordination devices [CCDs].” (p. 79, italics added). This dense formulation of culture consists of three variables—mind (proclivities), local practices (cultural paradigms) in specific societies, and established culture traditions known as CCDs, such as language, religion, norms, values, beliefs, attitudes, and so on. The wide margin of overlap and affinity between ours and Fiske’s three-component models of culture has been explored elsewhere (Sundararajan et al., 2024). In both models, the mind—functioning as either kin-based dispositions or proclivities—plays a central role in the maintenance and construction of culture.

What may be some of the advantages of our proposed mental model? First, in addition to the index of kinship intensity (Curtin et al., 2020) that measures the social-ecological dimension of culture, we have contributed another instrument to the toolbox of culture, by demonstrating how the rationality behind kin-based dispositions can be measured to make predictions about not only psychological traits, but also the connection between society and these psychological traits. Second, by decomposing culture into three components, our model approaches culture not as a piece of whole cloth - a package deal sealed by evolution so to speak - so much as a configuration of puzzle pieces that can sometimes fall apart. This approach is especially relevant in the globalization era in which cultural disruptions are widespread. A case in point is the investigation of three religious groups of an Indigenous population in Malaysia by Thong et al. (2023) who point out that, “The Traditional group [who resisted conversion to Islam or Christianity] managed to keep their strong-ties rationality intact, only to have an orphaned rationality, a rationality without its corresponding niche, as their preferred habitat, the forest, is continually being destroyed [by international logging companies]” (p. 9).

### 5.2 Limitations and future research directions

Due to its use of COVID-based items concerning lockdown, the *Strong-Ties Weak-Ties Rationality Scale* (STWTRS) may have limited shelf life in the post-COVID era. Continued assessment of its relevance is necessary, along with further development of a less time-sensitive version of STWTRS.

Our coverage of WT niche and rationality is scanty. By focusing on Americans with European ancestry only, we have neglected the mobile hunter-gathers and countries in the Middle East, both of which may represent less extreme forms of weak-ties than that of our non-Asian American sample. This lacuna needs to be filled in future studies with more widely varying types of societies and a more comprehensive test battery of WT values and beliefs.

Bourdieu (1977) makes a distinction between the logic of logic and the logic of practice. The de-contextualized analysis typical of cross-cultural analysis can only capture the logic of logic (i.e., the general principles) in ST and WT rationalities. To investigate the logic of practice—namely how ST and WT rationalities are being used by the cultural agents in specific social contexts—factor analysis for each sample needs to be conducted separately and the results for each society assessed holistically (see Ting et al., 2023).

Lastly, despite the novel approach in unpacking the moderating effects of the four societies, using regression analysis is still less than perfect because of the inherent assumption of independence among the predictors. If resources are available, researchers should consider testing the same hypotheses using a society-level sample size larger than 10, ideally larger than 30 (Hox et al., 2010). By so doing, we can delineate the effects of different levels and more accurately test the macro-level effects on the micro-level outcomes. This is the area of cross-cultural inquiry that awaits more researcher collaboration and joint endeavors (Smith and Bond, 2019).

## Data Availability

The datasets presented in this study can be found in online repositories. The names of the repository/repositories and accession number(s) can be found in the article/Supplementary material. Further inquiries can be directed to the corresponding author.

## References

[ref3] Bourdieu P. (1977). Outline of a theory of practice. (R. Nice, trans): Cambridge University Press (Original French edition 1972).

[ref4] Curtin C. M. Barrett H. C. Bolyanatz A. Crittenden A. N. Fessler D. M. T. Fitzpatrick S.. (2020). Kinship intensity and the use of mental states in moral judgment across societies. Evol. Hum. Behav. 41, 415–429. doi: 10.1016/j.evolhumbehav.2020.07.002

[ref5] Dalbert C. Lipkus I. Sallay H. Goch I. (2001). A just and an unjust world: structure and validity of different world beliefs. Pers. Individ. Differ. 30, 561–577. doi: 10.1016/s0191-8869(00)00055-6

[ref6] Enke B. (2019). Kinship, cooperation, and the evolution of moral systems. Q. J. Econ. 134, 953–1019. doi: 10.1093/qje/qjz001

[ref7] Fiske A. P. (2000). Complementarity theory: why human social capacities evolved to require cultural complements. Pers. Soc. Psychol. Rev. 4, 76–94. doi: 10.1207/S15327957PSPR0401_7, PMID: 15710561

[ref8] Fu P. P. Yukl G. (2000). Perceived effectiveness of influence tactics in the United States and China. Leadersh. Q. 11, 251–266. doi: 10.1016/s1048-9843(00)00039-4

[ref9] Graham J. Nosek B. A. Haidt J. Iyer R. Koleva S. Ditto P. H. (2011). Mapping the moral domain. J. Pers. Soc. Psychol. 101, 366–385. doi: 10.1037/a0021847, PMID: 21244182 PMC3116962

[ref10] Henrich J. (2020). The WEIRDest people in the world: How the west became psychologically peculiar and particularly prosperous: Farrar, Straus and Giroux.

[ref12] Hox J. Moerbeek M. van de Schoot R. (2010). Multilevel analysis: Routledge.

[ref13] Lim A. J. Lau C. Y. H. Cheng C.-Y. (2022). Applying the dual filial piety model in the United States: a comparison of filial piety between Asian Americans and Caucasian Americans. Front. Psychol. 12, 1–15. doi: 10.3389/fpsyg.2021.786609, PMID: 35185688 PMC8850268

[ref15] Misra G. Sundararajan L. Teo T. Ting R. S. K. Yang J. (2024). Decolonial research practices from an indigenous psychology perspective: critical contributions to knowledge. [Manuscript submitted for publication]. 10.1037/amp0001505 41379668

[ref16] Muthén B. Asparouhov T. (2014). IRT studies of many groups: the alignment method. Front. Psychol. 5:978. doi: 10.3389/fpsyg.2014.00978, PMID: 25309470 PMC4162377

[ref17] Ralston D. A. Terpstra-Tong J. L. Y. Trevino L. Caprar D. Froese F. Furrer O.. (2023). “A 40-society perspective on the impact of microculture cohorts on preferences for type of organizational culture” in Paper presented at the annual meeting of the academy of international business (Miami).

[ref19] Saroglou V. Delpierre V. Dernelle R. (2004). Values and religiosity: a meta-analysis of studies using Schwartz’s model. Pers. Individ. Differ. 37, 721–734. doi: 10.1016/j.paid.2003.10.005

[ref20] Schulz J. F. Bahrami-rad D. Beauchamp J. P. Henrich J. (2019). The church, intensive kinship, and global psychological variation. Science 366:eaau5141. doi: 10.1126/science.aau5141, PMID: 31699908

[ref21] Smith P. B. Bond M. H. (2019). Cultures and persons: characterizing national and other types of cultural difference can also aid our understanding and prediction of individual variability. Front. Psychol. 10:2689. doi: 10.3389/fpsyg.2019.02689, PMID: 31849785 PMC6901915

[ref22] Sundararajan L. (2020). Strong-ties and weak-ties rationalities: toward an expanded network theory. Rev. Gen. Psychol. 24, 134–143. doi: 10.1177/1089268020916438

[ref23] Sundararajan L. Ting R. S.-K. Hsieh S.-K. Kim S.-H. (2022). Religion, cognition, and emotion: what can automated text analysis tell us about culture? Humanist. Psychol. 50, 213–233. doi: 10.1037/hum0000201

[ref24] Sundararajan L. Yeh K. -H. (2022). Strong ties and weak ties rationality: theory and scale development. Integr. Psychol. Behav. Sci. 56, 405–419. doi: 10.1007/s12124-021-09645-5, PMID: 34478020 PMC8413355

[ref25] Sundararajan L. Yeh K. -H. Ting R. S. -K. Terpstra-Tong J. Bond M. H. (2024). Toward culture as an ontological universe: Implications for cultural loss and interventions in the globalizing era. [Manuscript submitted for publication].

[ref26] Talhelm T. Zhang X. Oishi S. Shimin C. Duan D. Lan X.. (2014). Large-scale psychological differences within China explained by rice versus wheat agriculture. Science 344, 603–608. doi: 10.1126/science.1246850, PMID: 24812395

[ref27] Thong J. J.-A. Ting R. S.-K. Jobson L. Sundararajan L. (2023). “In the wake of religious conversions: Differences in cognition and emotion across three religious communities of an indigenous tribe in Malaysia” in Psychology of Religion and Spirituality. Advance online publication

[ref28] Ting R. S.-K. Zay Hta M. K. Yeh K.-H. Ng V. H. C. Liu C. Xie Z. Y.. (2023). Mapping culture and rationality across four countries: expanding the conceptual horizons of strong-ties and weak-ties rationality. The Humanistic Psychologist. doi: 10.1037/hum0000339

[ref29] Todd P. M. Gigerenzer G. The ABC Research Group (2012). Ecological rationality: Intelligence in the world. UK: Oxford University Press.

[ref30] Van de Vijver F. J. R. Leung K. (2011). Equivalence and bias: a review of concepts, models, and data analytic procedures. In Matsumoto D. Vijver F. J. R. Van de (Eds.), Cross-cultural research methods in psychology (pp. 17–45). Cambridge University Press.

[ref31] Welzel C. Inglehart W. R. (2010). Agency, values, and well-being: a human development model. Soc. Indic. Res. 97, 43–63. doi: 10.1007/s11205-009-9557-z, PMID: 20390031 PMC2848347

[ref32] Wu M. S. Yan X. Zhou C. Chen Y. Li J. Zhu Z.. (2011). General belief in a just world and resilience: evidence from a collectivistic culture. Eur. J. Pers. 25, 431–442. doi: 10.1002/per.807

[ref33] Wu C.-W. Yeh K.-H. (2021). Self-sacrifice is not the only way to practice filial piety for Chinese adolescents in conflict with their parents. Front. Psychol. 12:661335. doi: 10.3389/fpsyg.2021.661335, PMID: 34054663 PMC8160098

[ref34] Yeh K.-H. Bedford O. (2003). A test of the dual filial piety model. Asian J. Soc. Psychol. 6, 215–228. doi: 10.1046/j.1467-839x.2003.00122.x

[ref35] Yeh K.-H. Sundararajan L. Ting R. S.-K. Liu C. Liu T. Zhang K. (2022). A cross-cultural study of strong ties and weak ties rationalities: toward an ontological turn in psychology. Humanist. Psychol. 51, 235–259. doi: 10.1037/hum0000284

[ref36] Yeh K.-H. Yi C.-C. Tsao W.-C. Po-San Wan P.-S. (2013). Filial piety in contemporary Chinese societies: a comparative study of Taiwan, Hong Kong, and China. Int. Sociol. 28:296. doi: 10.1177/0268580913484345

[ref37] Zay Hta M. K. Ting R. S.-K. Jones L. (2024). The relationship between strong-ties weak-ties rationality and COVID-19 public stigma: a cross-cultural study of Malaysia and Australia. Int. J. Psychol. Advance online publication. doi: 10.1002/ijop.13155, PMID: 38843891

