# Supplementary material for: Strong-ties and weak-ties rationalities: toward a mental model of the consequences of kinship intensity
Source: Front Psychol. 2024 Nov 13;15:1476018. doi: 10.3389/fpsyg.2024.1476018 (PMC11603357; doi:10.3389/fpsyg.2024.1476018)
Supplement: Supplementary file 1 [file Table_1.DOCX]

**Supplementary Materials**

**Appendix S1**

**Procedure of Data Collection by Society**

*China sample*: In China, 364 participants were recruited through online platforms, with the majority through the Star Network Platform (https://www.wjx.cn), the most commonly used online questionnaire platform in mainland China, and the rest through the WeChat platform. The default recruitment setting for participant ethnicity was Han and minority samples were excluded. The participants were predominantly community residents, working class and student groups. Compensation for participation consisted of cash rewards, averaging about 8 yuan (about USD1.0). The difference in the reward amounts across platforms is mainly due to different practices adopted by the platforms. Data from four participants who failed to pass the filter item checks were dropped. After data cleaning, we obtained a final usable sample of 359 for China.

*Taiwan sample*. A total of 303 participants were recruited from Surveycake platform online.
Fourteen were excluded due to either completing the questionnaire in less than 400 seconds or failing to meet the filter criteria. The remaining 289 participants were included in the analysis. The participants spent about 20 minutes answering the survey, which began with a consent form outlining the purpose of this study. Upon completion, they received compensation equivalent to two to three US dollars.

*India sample*: We recruited participants to join from workgroups, and academic settings through social networking. They were volunteers and received no compensation. Initially, we received a total of 396 valid samples through social networking platforms and classrooms in colleges from North and West India over three waves between the end of 2021 and May, 2022. Of the initial pool, 131 were excluded due to either completing the questionnaire in less than 400 seconds or failing to pass the filter criteria.

*The U.S. sample.* Participants were recruited through Amazon’s Mechanical Turk. We asked three screening questions to determine participants’ eligibility for the study: Aged 20 years or older; being born and currently residing in the United States with an American citizenship; and having continuously lived in the United States during the past three years without spending over three months abroad. Those with dual citizenship were excluded. The survey took about 20 minutes to complete, and participants were compensated $2 for their participation. Initially, a total of 702 survey responses were collected. After data checking, a total of 153 responses were excluded from data analysis. The remaining valid sample of 549 participants who passed the screening was retained. All met the inclusion criteria of spending at least 400 seconds in completing the questionnaire. After breaking down by race, 169 participants were of Asian American descent, and 379 were non-Asians (86% white, 6% black, 5% Hispanic, 0.26% Middle Eastern/North African, 0.53% Pacific Islander/Native Hawaiian). In this study, we only included the data of non-Asia Americans in the analysis.

**Appendix S2**

**Alignment Results of Unidimensional Scales**

**Approximate Measurement Invariance**

| Notes: Numeric values refer to non-invariant societies (1=Taiwan; 2=China; 3=India; 4=the U.S.)  **Strong ties Rationality** | | | | | |
| --- | --- | --- | --- | --- | --- |
| Intercepts/Thresholds | |  |  |  |  |
| STWT04 1 2 (3) 4 | |  |  |  |  |
| STWT05 1 2 3 4 | |  |  |  |  |
| STWT09 1 2 3 (4) | |  |  |  |  |
| STWT16 1 (2) 3 4 | |  |  |  |  |
| STWT17 1 2 3 4 | |  |  |  |  |
| STWT20 1 2 3 (4) | |  |  |  |  |
|  |  |  |  |  |  |
| Loadings for ST | |  |  |  |  |
| STWT04 1 2 3 4 | |  |  |  |  |
| STWT05 1 (2) 3 4 | |  |  |  |  |
| STWT09 1 2 3 4 | |  |  |  |  |
| STWT16 1 2 3 4 | |  |  |  |  |
| STWT17 1 (2) 3 4 | |  | Non-invariance rate | |  |
| STWT20 1 2 3 4 | |  | 12.50% |  |  |

**Weak-ties Rationality**

| Intercepts/Thresholds | |  |  |  |
| --- | --- | --- | --- | --- |
| STWT02 1 2 3 4 | |  |  |  |
| STWT03 1 2 3 4 | |  |  |  |
| STWT06 1 2 3 (4) | |  |  |  |
| STWT07 1 2 3 4 | |  |  |  |
| STWT10 1 2 3 (4) | |  |  |  |
| STWT11 1 2 3 4 | |  |  |  |
| STWT14 1 2 3 (4) | |  |  |  |
| STWT15 1 (2) 3 4 | |  |  |  |
| STWT18 1 (2) 3 4 | |  |  |  |
| STWT19 1 (2) 3 4 | |  |  |  |
|  |  |  |  |  |
| Loadings for WT | |  |  |  |
| STWT02 1 2 3 4 | |  |  |  |
| STWT03 1 2 3 4 | |  |  |  |
| STWT06 1 2 3 4 | |  |  |  |
| STWT07 1 2 3 4 | |  |  |  |
| STWT10 1 2 3 4 | |  |  |  |
| STWT11 1 2 3 4 | |  |  |  |
| STWT14 1 2 3 4 | |  |  |  |
| STWT15 1 2 3 4 | |  |  |  |
| STWT18 (1) 2 3 4 | |  | Non-invariance rate | |
| STWT19 1 2 3 (4) | |  | 10% |  |

**Reciprocal Filial Piety**

| Intercepts/Thresholds | | | | | | |  | | | |  | |  | |
| --- | --- | --- | --- | --- | --- | --- | --- | --- | --- | --- | --- | --- | --- | --- |
| FP01 1 2 3 4 | | | | | |  |  | | | |  | |  | |
| FP03 1 2 3 4 | | | | | |  |  | | | |  | |  | |
| FP05 1 (2) 3 4 | | | | | |  |  | | | |  | |  | |
| FP07 1 2 3 4 | | | | | |  |  | | | |  | |  | |
| FP09 1 2 3 (4) | | | | | |  |  | | | |  | |  | |
| FP11 1 2 3 4 | | | | | |  |  | | | |  | |  | |
| FP13 1 2 3 4 | | | | | |  |  | | | |  | |  | |
|  | | | | | |  |  | | | |  | |  | |
| Loadings for RFP | | | | | |  |  | | | |  | |  | |
| FP01 1 2 3 (4) | | | | | |  |  | | | |  | |  | |
| FP03 1 2 3 4 | | | | | |  |  | | | |  | |  | |
| FP05 1 2 3 (4) | | | | | |  |  | | | |  | |  | |
| FP07 1 2 3 4 | | | | | |  |  | | | |  | |  | |
| FP09 1 2 3 4 | | | | | |  |  | | | |  | |  | |
| FP11 1 2 3 4 | | | | | |  |  | | | |  | |  | |
| FP13 1 2 (3) 4 | | | | | |  |  | | | |  | |  | |
| Non-invariance rate | | | | | |  |  | | | | | |  | |
| 10.73% | | | | | |  |  | | | |  | |  | |
| **Authoritarian Filial Piety** | | | | | |  |  | | | |  | |  | |
| Intercepts/Thresholds | | |  | | | | | |  | | |  |  |  |
| FP02 1 2 3 4 | | |  | | | | | |  | | |  |  |  |
| FP04 1 2 3 (4) | | |  | | | | | |  | | |  |  |  |
| FP08 1 2 3 4 | | |  | | | | | |  | | |  |  |  |
| FP10 1 2 3 4 | | |  | | | | | |  | | |  |  |  |
| FP12 1 2 3 4 | | |  | | | | | |  | | |  |  |  |
| FP14 1 2 (3) 4 | | |  | | | | | |  | | |  |  |  |
| FP16 1 2 3 (4) | | |  | | | | | |  | | |  |  |  |
|  |  | |  | | | | | |  | | |  |  |  |
| Loadings for RFP | | |  | | | | | |  | | |  |  |  |
| FP02 1 2 (3) 4 | | |  | | | | | |  | | |  |  |  |
| FP04 1 2 3 4 | | |  | | | | | |  | | |  |  |  |
| FP08 1 2 3 4 | | |  | | | | | |  | | |  |  |  |
| FP10 1 2 3 4 | | |  | | | | | |  | | |  |  |  |
| FP12 1 2 3 4 | | |  | | | | | |  | | |  |  |  |
| FP14 1 2 3 4 | | |  | | | | | |  | | |  |  |  |
| FP16 1 2 3 (4) | | |  | | | | | |  | | |  |  |  |
|  |  | | Non-invariance rate | | | | | | | | |  |  |  |
|  |  | | 8.93% | | | | | |  | | |  |  |  |
|  |  | |  | | | | | |  | | |  |  |  |
| **Belief in a Just World** | | | | | | | | |  |  |  |  |  |  |
| Intercepts/Thresholds | | |  | | | | | |  |  |  |  |  |  |
| JWS01 1 2 3 4 | | |  | | | | | |  |  |  |  |  |  |
| JWS02 1 (2) 3 4 | | |  | | | | | |  |  |  |  |  |  |
| JWS04 1 2 3 4 | | |  | | | | | |  |  |  |  |  |  |
| JWS05 1 2 3 (4) | | |  | | | | | |  |  |  |  |  |  |
| JWS06 1 2 3 4 | | |  | | | | | |  |  |  |  |  |  |
|  |  | |  | | | | | |  |  |  |  |  |  |
| Loadings for JWB | | |  | | | | | |  |  |  |  |  |  |
| JWS01 (1) 2 3 4 | | |  | | | | | |  |  |  |  |  |  |
| JWS02 1 2 3 4 | | |  | | | | | |  |  |  |  |  |  |
| JWS04 1 2 3 4 | | |  | | | | | |  |  |  |  |  |  |
| JWS05 1 2 3 4 | | |  | | | | | |  |  |  |  |  |  |
| JWS06 1 2 3 4 | | |  | | | | | |  |  |  |  |  |  |
|  |  | | 7.50% | | | | | |  |  |  |  |  |  |
| **Belief in an Unjust World** | | |  | | | | |  |  |  |  |  |  |  |
| Intercepts/Thresholds | | |  | | | | |  |  |  |  |  |  |  |
| JWS07 1 2 3 4 | | |  | | | | |  |  |  |  |  |  |  |
| JWS08 1 2 3 4 | | |  | | | | |  |  |  |  |  |  |  |
| JWS09 (1) 2 3 4 | | |  | | | | |  |  |  |  |  |  |  |
| JWS10 1 2 3 4 | | |  | | | | |  |  |  |  |  |  |  |
|  | | |  | | | | |  |  |  |  |  |  |  |
| Loadings for UJWB | | |  | | | | |  |  |  |  |  |  |  |
| JWS07 1 2 3 4 | | |  | | | | |  |  |  |  |  |  |  |
| JWS08 1 2 3 4 | | |  | | | | |  |  |  |  |  |  |  |
| JWS09 1 2 3 4 | | |  | | | | |  |  |  |  |  |  |  |
| JWS10 1 2 3 4 | | |  | | | | |  |  |  |  |  |  |  |
|  | | | 3.13% | | | | |  |  |  |  |  |  |  |
| **Dharma** | | |  | | | | |  |  |  |  |  |  |  |
| Intercepts/Thresholds | |  | | | | | | | | | |  | |  |
| MFQ05 1 2 3 4  MFQ10 1 2 3 4  MFQ11 1 2 3 (4)  MFQ15 1 (2) 3 4  MFQ16 1 2 3 4  Loadings for DHARMA    MFQ05 1 2 3 4  MFQ10 1 2 3 4  MFQ11 1 2 3 4  MFQ15 1 2 3 4  MFQ16 1 2 3 4 | | | | | | | | |  | | |  |  |  |
|  |  |  |  |  |  |  |  |  |  | | |  |  |  |
|  |  |  |  |  |  |  |  |  |  | | |  |  |  |
|  |  |  |  |  |  |  |  |  |  | | |  |  |  |
|  |  |  |  |  |  |  |  |  |  | | |  |  |  |
|  |  |  |  |  |  |  |  |  |  | | |  |  |  |
|  |  |  |  |  |  |  |  |  |  | | |  |  |  |
|  |  |  |  |  |  |  |  |  |  | | |  |  |  |
|  |  |  |  |  |  |  |  |  |  | | |  |  |  |
|  |  |  |  |  |  |  |  |  |  | | |  |  |  |
|  |  |  |  |  |  |  |  |  |  | | |  |  |  |
|  |  |  |  |  |  |  |  |  |  | | |  |  |  |
|  |  |  |  |  |  |  |  |  |  | | |  |  |  |
|  |  |  |  |  |  |  |  |  |  | | |  |  |  |
|  | 5.00% | | |  |  | | | | |  |  |  |  |  |
|  |  | |  | | | | | |  | | |  |  |  |

**Appendix S3**

**Final Measures used in the Study**

**Belief in a just world**

1. I think basically the world is a just place.
2. I believe that, by and large, people get what they deserve.
3. I am convinced that in the long run people will be compensated for injustices.
4. I firmly believe that injustices in all areas of life (e.g., professional, family, politic) are the exception rather than the rule
5. I think people try to be fair when making important decisions.

**Belief in an unjust world**

1. A lot of people suffer an unjust fate.
2. I feel that even important decisions are often unfair.
3. I basically believe the world is an unjust place.
4. I feel that people won’t be compensated for injustices too often.

**Dharma**

1. People should not do things that are disgusting, even if no one is harmed.
2. Men and women each have different roles to play in society.
3. I would call some acts wrong on the grounds that they are unnatural.
4. If I were a soldier and disagreed with my commanding officer’s orders, I would obey anyway because that is my duty.
5. Chastity is an important and valuable virtue.

**Filial Piety**

*Authority filial piety*

1. Take my parents’ suggestions even when I do not agree with them.
2. Let my income be handled by my parents before marriage.
3. Give up my aspirations to meet my parents’ expectations.
4. Do whatever my parents ask right away.
5. Avoid getting married to someone my parents dislike.
6. Have at least one son for the succession of the family name.
7. Living with the husband's parents after being married.

*Reciprocal filial piety*

1. Be concerned about my parents’ health.
2. Talk frequently with my parents to understand their thoughts and feelings.
3. Be concerned about my parents’ general well-being.
4. Be concerned about my parents, as well as understand them.
5. Support my parents’ livelihood to make their lives more comfortable.
6. Be grateful to my parents for raising me.
7. Attend the parent’s funeral no matter how far away I live.

**Strong Ties Weak Ties Rationality Scale**

*Strong-ties rationality*

1. When an infectious disease is spreading, the most important strategy for prevention is to completely separate the infected person from the rest of the population. Otherwise the whole community may get infected.
2. Saying positive things creates positive energy that can spread like fresh air to benefit the whole community.
3. Spreading rumours is like polluting the air, it poisons all who are exposed to it.
4. Optimism is very important during a lockdown since it spreads positive energy to benefit the whole society.
5. It is important not to complain or gripe during a lockdown, because these words spread negative energy and can hurt many people around you.

*Weak-ties rationality*

1. Transmission of the coronavirus works like match sticks. If one matchstick is burning, the next match stick will catch fire only if it is too close to it. If the two match sticks are far apart, the second one will not catch fire.
2. When an infectious disease is spreading, the most important strategy for prevention is to keep a distance of 6 feet from each other.
3. People who do not wear face masks during the pandemic are not necessarily bad people, but they should not be allowed into stores.
4. The key to successful prevention of personal infection lies in maintaining safe behaviour: hand washing, mask wearing, and social distancing.
5. In implementing a lockdown, it is important to balance the interests of the group with the interests of individuals, so that one is not served at the expense of the other.
6. Fact-checking is effective in debunking false information.
